# Malignancy and viral infections in Sub-Saharan Africa: A review

**DOI:** 10.3389/fviro.2023.1103737

**Published:** 2023-03-06

**Authors:** Mahamadou Diakite, Kathryn Shaw-Saliba, Chuen-Yen Lau

**Affiliations:** 1 University Clinical Research Center, University of Sciences, Techniques, and Technologies, Bamako, Mali; 2 Collaborative Clinical Research Branch, Division of Clinical Research, National Institute of Allergy and Infectious Diseases (NIAID), National Institutes of Health (NIH), Bethesda, MD, United States; 3 HIV Dynamics and Replication Program, National Cancer Institute (NCI), National Institutes of Health (NIH), Bethesda, MD, United States

**Keywords:** virus, infection, malignancy, oncogenesis, Sub-Saharan Africa (SSA)

## Abstract

The burden of malignancy related to viral infection is increasing in Sub-Saharan Africa (SSA). In 2018, approximately 2 million new cancer cases worldwide were attributable to infection. Prevention or treatment of these infections could reduce cancer cases by 23% in less developed regions and about 7% in developed regions. Contemporaneous increases in longevity and changes in lifestyle have contributed to the cancer burden in SSA. African hospitals are reporting more cases of cancer related to infection (e.g., cervical cancer in women and stomach and liver cancer in men). SSA populations also have elevated underlying prevalence of viral infections compared to other regions. Of 10 infectious agents identified as carcinogenic by the International Agency for Research on Cancer, six are viruses: hepatitis B and C viruses (HBV and HCV, respectively), Epstein-Barr virus (EBV), high-risk types of human papillomavirus (HPV), Human T-cell lymphotropic virus type 1 (HTLV-1), and Kaposi’s sarcoma herpesvirus (KSHV, also known as human herpesvirus type 8, HHV-8). Human immunodeficiency virus type 1 (HIV) also facilitates oncogenesis. EBV is associated with lymphomas and nasopharyngeal carcinoma; HBV and HCV are associated with hepatocellular carcinoma; KSHV causes Kaposi’s sarcoma; HTLV-1 causes T-cell leukemia and lymphoma; HPV causes carcinoma of the oropharynx and anogenital squamous cell cancer. HIV-1, for which SSA has the greatest global burden, has been linked to increasing risk of malignancy through immunologic dysregulation and clonal hematopoiesis. Public health approaches to prevent infection, such as vaccination, safer injection techniques, screening of blood products, antimicrobial treatments and safer sexual practices could reduce the burden of cancer in Africa. In SSA, inequalities in access to cancer screening and treatment are exacerbated by the perception of cancer as taboo. National level cancer registries, new screening strategies for detection of viral infection and public health messaging should be prioritized in SSA’s battle against malignancy. In this review, we discuss the impact of carcinogenic viruses in SSA with a focus on regional epidemiology.

## Graphical Abstract

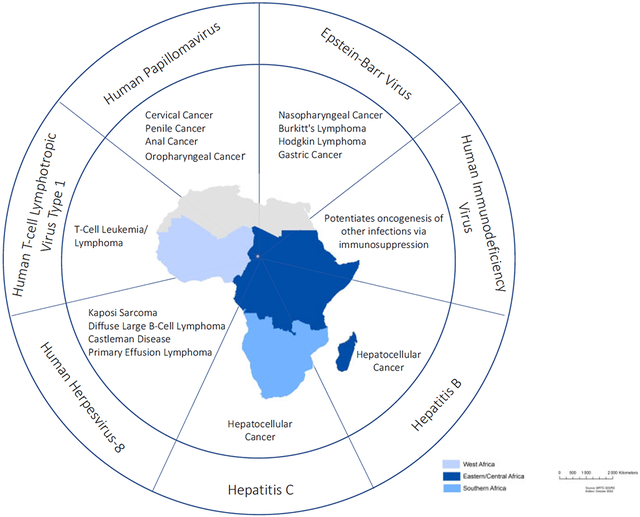

Oncogenic Viruses in Sub-Sahara Africa. The outer circle shows the six viruses classsified as oncogenic by the international Agency for Research on Cancer plus Human Immunodeficiency Virus, which potentiates oncogenesis. Examples of malignancies attributable to each virus are shown in the inner circle.

## Introduction

According to the World Health Organization (WHO), approximately six of every 100 cancer deaths in developed countries are linked to infection (1). Malignancies related to infection are a much more serious problem in developing regions, particularly Africa. For example, cervical cancer and non-Hodgkin lymphoma associated with human papillomavirus (HPV) and Epstein-Barr virus (EBV) infections, respectively, are disproportionately common in Africa (2). Malignancy due to infection is preventable. Thus, oncogenic viruses are an important cancer control target.

Africa hosts tremendous genetic diversity (3, 4) in conjunction with high infectious disease burden and socio-cultural diversity. This combination presents great opportunity for understanding epidemiology and pathogenesis of oncogenic viruses, but also presents challenges for prevention and control of associated malignancy. Yet the study of viral oncogenesis specific to SSA (West, Central, Eastern and Southern Africa) is relatively young. The earliest arguments linking viruses and cancer have their official origins in the discovery of two bird viruses. The first, avian leukemia virus, was discovered by Vilhelm Ellerman and Olaf Bang, two biologists at the University of Copenhagen in 1908 (5, 6). Leukemias were not considered malignancies at that time, so this discovery was overshadowed by contemporaneous work by Francis Peyton Rous in the United States. In 1911, Rous described a form of avian sarcoma, a well-known tumor, and demonstrated its association with Rous sarcoma virus (7). These earliest oncogenic viruses were retroviruses (8), the study of which laid the foundation for our current understanding of cancer.

Cancer is the second leading cause of death worldwide (after ischemic heart disease). As of 2020, the global burden of cancers caused by infection was estimated at 15.4%. The estimate is over 30% for SSA, making infections amongst the most important causes of cancer in the region (9, 10). Cancer-causing infections are responsible for approximately 30% of cancer cases in low- and lower-middle-income countries. Infection as an etiology of cancer may be increasing, as only 13% of cancers diagnosed in 2018 globally were considered attributable to carcinogenic infections, including Helicobacter pylori, HPV, HBV, HCV, and EBV (2, 11).

Characterizing the link between infection and cancer is an active area of research, particularly in Africa. Elucidating the relationship in SSA is exceptionally challenging due to a high frequency of co-infections. Furthermore, seroprevalence estimates are difficult due to regional heterogeneity of patient populations, size of studies and serodiagnostic methods employed. Temporal uncertainties also complicate the picture, as infections detectable at time of cancer diagnosis may differ from those which played a causal role. Additionally, oncogenic viruses appear to be the etiology of malignancy in a minority of cases, with genetics and lifestyle factors playing a key role.

We will discuss the oncogenic viruses HBV, HCV, EBV, HPV, HTLV-1, HHV-8, HIV, and their impact in SSA in this review. While we will focus on epidemiology, pathogenesis, socio-cultural contributors, and suggestions for additional investigation will also be addressed. Understanding these viruses and their respective malignancies will facilitate effective public policy and advancement of prevention and treatment programs in SSA.

## Hepatitis B virus

### HBV genomic organization and protein products

HBV is the smallest human DNA virus with 3,200 base pairs. Its double-stranded circular genome contains four overlapping open reading frames (ORFs) and encodes 4 genes: C, S, P and X. The C gene, with a pre-C zone, encodes the 21 kDa core (HBc) antigen (Ag) and HBeAg. The S gene, with a pre-S1 zone and a pre-S2 zone, encodes the surface antigen (HBsAg) (12). HBsAg exists in small 24 kDa, medium 33kDa, and large 39 kDa forms depending on whether it derives from expression of S, pre-S2 + S, or pre-S1 + pre-S2 + S genes. The P gene encodes the 90 kDa DNA polymerase, which serves as a classic DNA-dependent DNA polymerase, a retrotranscriptase, and has RNase H activity. The X gene encodes X protein (HBx), which performs transactivating functions, regulates the viral lifecycle and is required for replication (13). HBx is likely involved in HBV carcinogenesis (14, 15). The HBV genome also includes four promoters, two enhancer elements (EN1 and EN2), a polyadenylation site for viral RNA transcription, and several cis-acting signals for DNA replication.

To optimize its limited capacity, HBV’s genes overlap. The P gene, the longest of the four, comprises three-quarters of the genome and overlaps entirely with the S gene and partially with the C and X genes (16). All HBV RNA transcripts terminate at the same polyadenylation site located in the C ORF (17). A posttranslational *cis* regulatory element (PRE), which overlaps with part of the X ORF, promotes nuclear export of unspliced HBV RNA to the cytoplasm (18).

This strictly human Hepadnavirus consists of an envelope bearing HBsAg with an interior nucleocapsid. HBV viral glycoproteins (HBsAg) constitute the envelope surrounding the viral capsid in mature virions; HBsAg contains the main HBV antigenic domains and the attachment site to target hepatocytes (19). HBcAg forms the core or capsid and is expressed on the surface of hepatocytes, where it induces cytolysis *via* CD8^+^ T lymphocytes. Unlike HBsAg, it does not appear in serum. Conversely, the smaller HBeAg is present in the serum during active viral multiplication (20). Expression of 10 amino acids from the pre-C region on HBeAg enable its passage through the reticuloendothelial system and excretion in serum (21). HBsAg, HBcAg and HBeAg and their respective antibodies are markers of HBV infection status.

HBsAg has at least 5 main antigenic sites. The major determinant (a) is common to all strains and corresponds to production of HBsAb. There are also two pairs of mutually exclusive subtype determinants which define 4 subtypes of HBsAg: adw, ayw, adr and ayr (22). The HBsAg subtypes have geographical specificity. For example, adw predominates in northern Europe, North and South America, and Australia, while ayr is found in northern and eastern Africa, the eastern Mediterranean, eastern Europe, northern and central Asia, and India. HBV genotyping now allows distinguishing genotypes from A to H by PCR. Characteristics of HBV genotypes and subtypes have been used to investigate maternal transmission, familial clustering, and geographic distribution of HBV strains (23).

HBV was identified in 1967. Since then, it has come to be recognized as a cause of hepatitis, cirrhosis, and hepatocellular carcinoma. HBV is a leading cause of malignancy in SSA.

### HBV prevalence and distribution in SSA

Approximately 296 million people worldwide are living with HBV and 820,000 die annually from related complications (24). Though effective vaccination has reduced incidence, HBV affects 8%-18% of the SSA population, with 5-10% of the adult population being chronically infected (25). Propensity for chronicity (~2%) makes it the leading cause of cirrhosis and hepatocellular carcinoma in the region. Most chronic HBV infections result from perinatal transmission (26). The epidemiology of HBV in Africa is also intertwined with that of HIV/AIDS, given the overlap in risk factors.

HBV genotypes A, D and E are circulating in Africa. Genotype A is predominant in southern Africa. African subtypes A1 and Aa are associated with early HBe seroconversion and lower viral loads compared to European subtypes A2 and Ae. Genotype D prevails in North Africa. Genotype D is also associated with early HBe seroconversion and high rates of HBeAg negative active hepatitis. Genotype E is observed in the West and Central African regions of SSA (27).

HBV is transmitted *via* bodily fluids parenterally, sexually, and perinatally. Vertical infection is the most common route of transmission in high endemicity areas, including in SSA. Perinatal transmission is associated with greatest risk of chronic carriage and is more common the later a woman is infected during pregnancy (28, 29). Despite availability of accurate testing and an efficacious vaccine, access to affordable diagnostics and vaccination coverage remain suboptimal in SSA (30, 31). Regional awareness of HBV infection status remains very low, further fueling spread of infection and unchecked disease progression (32).

HBV has a broad spectrum of clinical manifestations including asymptomatic disease, fulminant hepatitis, and hepatocellular carcinoma (HCC). Disease can be acute or chronic, though acute disease is typically self-limited (33). Chronic disease is much more common after perinatal transmission versus acquisition in adulthood. When left untreated, HBV infection can engender liver fibrosis, cirrhosis, and eventually liver cancer. Low rates of detection in SSA result in untreated infections and higher rates of HCC. The confluence of pathogenic mechanisms and epidemiologic risks have made HBV the leading cause of HCC in SSA.

### HBV-associated malignancies

HBV leads to HCC typically after many years of infection and in the setting of fibrosis. Longitudinal studies of untreated people with chronic HBV show a 5-year risk for developing cirrhosis of 8-20% (34-38). Amongst those with cirrhosis, the annual risk of hepatic decompensation is approximately 20% and the annual incidence of HBV-related HCC ranges from <1%-5% (39). Untreated decompensated cirrhosis carries a poor prognosis, with survival of 15-40% at five years (38-40). Several host and viral factors, particularly co-infections with HIV, HCV and hepatitis D virus (HDV), as well as alcohol consumption and underlying liver disease, increase the rate of disease progression and the risk of developing HCC (35-38, 41). Other risk factors include age, sex, serum alanine aminotransferase (ALT) levels, serum HBV DNA levels, and HBeAg levels (42).

Integration of HBV DNA into host chromosomes has been found in >80% of HBV associated HCC (43, 44). Specific integration hotspots have been identified (45), particularly within the X gene (46-48). HBV DNA integration occurs early in infection (49), persists, and can activate or disrupt gene expression to promote chromosomal instability and development of cancer, metastases, and angiogenesis (50).

Low engagement in routine preventive care in SSA means that disease progression typically goes unchecked. Approximately 80% of newly diagnosed HBV in Africa is linked to chronic disease (51), and hence risk of HCC. Males with HBV are 5-7 times more likely than females to progress to HCC (52), which is in part attributable to positive feedback through the androgen receptor (53, 54) and differing levels of chronic liver inflammation. Further epidemiologic characterization of the HBV epidemic in SSA is needed to determine if HCC associations will reflect those seen in other populations.

### Future directions

The true extent of HBV infection in the African region has not been fully characterized. National and subnational level data is lacking; hepatitis surveillance programs are performing poorly; and thus (55), priorities are difficult to determine for targeted measures and resource allocation (56, 57). Several approaches for prevention and control of viral hepatitis, integration of HBV vaccination into national immunization programs, and routine vaccination of healthcare workers have been proposed (58, 59). With increasing accessibility of HBV vaccines, more affordable diagnostics, and effective antiviral drugs, control of SSA’s HBV epidemic is becoming more feasible.

Addressing the interplay between HBV and HIV co-infection will continue to be important, as both infections impact SSA and risk factors overlap (60). Co-infection can accelerate progression of both infections and inhibit HBV clearance *via* effects on the host immune response, thus contributing to increased rates of cirrhosis and hepatocellular carcinoma (61-71). All people with HBV should be screened for HIV and vice versa. Accurate assessment of infection will enable appropriate use of antivirals. For example, HIV/HBV co-infected people should be treated with two anti-retrovirals that suppress HBV as part of the HIV treatment regimen to minimize progression and transmission of both infections (72). Detection and management of co-infections should be integrated into current public health systems.

HBV prevention activities in Africa are improving but remain limited. Full implementation of perinatal HBV vaccination has been slow in SSA (73). HBV vaccination is a safe and effective HCC prevention method and must be prioritized (33, 74). Scalable screening programs are also lacking in SSA, where <5% of people with chronic hepatitis are aware of their HBV status (30, 55, 75, 76). Broad implementation of screening should be accompanied by improved access to indicated follow up and management when infections are detected (77, 78). Training of healthcare providers and overcoming structural barriers that perpetuate vulnerability, marginalization of affected populations and inequitable access to services will be critical. Although many SSA countries do not screen or only partially screen donated blood products, transfusion surveillance (79) indicates that broader screening of blood products will be needed to reduce transfusion associated transmission (80, 81-83).

SSA countries should establish a rigorous viral hepatitis surveillance system. Research on prevention and management strategies that consider SSA biology and socio-economic structure should be expanded to complement surveillance. Table 1 shows key information on factors related to acquisition of infection, disease course, and prevention strategies for HBV.

## Hepatitis C virus

### HCV genomic organization and protein products

Hepatitis C Virus (HCV) is single positive stranded RNA virus in the family *Flaviviridae* (84). It contains a 5’ non-translated region (NTR), a long open reading frame encoding a polyprotein, and a 3’ NTR. The polyprotein is cleaved by host enzymes and viral proteases into >10 products. Core (C) is an RNA binding protein which forms the nucleocapsid. E1 and E2 are highly glycosylated type 1 transmembrane proteins which comprise stable heterodimers embedded into the lipid envelope around the viral nucleocapsid. P7 is a small hydrophobic peptide which may be a virioporin involved in release of virions from infected cells. The nonstructural proteins NS2 - NS5B are primarily required for multiplication of viral RNA. NS2 and the amino terminal domain of NS3 constitute the NS2/3 autoproteinase responsible for NS2/3 junction cleavage. NS3 has three different enzymatic functions (85). The amino-terminal domain forms a serine-type proteinase responsible for processing at the carboxy terminal of NS3 and the carboxy terminal domain carries NTPase/helicase activities.

While hepatocytes are the primary host cell, replication also occurs in peripheral blood mononuclear cells (PBMCs) and B and T cell lines. When HCV binds the host cell, viral RNA is released from the nucleocapsid into the cytoplasm, the genome is translated, and the resulting polyprotein is cleaved into individual proteins. These proteins form a higher-order multi-protein complex tightly associated with intracellular membranes. Within this complex, the positive strand RNA genome is copied into a negative strand RNA intermediate that serves as a template for synthesis of positive strand progeny (84). These positive strands may be used for synthesis of new negative strands, translated, or encapsulated into virions.

HCV has six major genotypes, though other lesser contributors have been identified. Each genotype can have multiple subtypes, for example 1a, 1b, 2a and 2b (85, 86). There is high variability of genotypes across SSA, particularly in g4 and g7 strains (87). Furthermore, the ΔG frame shift allele which encodes an open reading frame of IFN-λ4 is a major variant in Africans and influences clinical outcomes through modulation of IFN stimulated gene expression (88). Though initially studied with relation to IFN-based treatment, this allele is also associated with delayed response to currently used DAAs (89). Genotypic predominance not only varies geographically but is also temporally dynamic in association with population interactions.

### HCV prevalence and distribution in SSA

Prevalence of chronic HCV varies from 0.2% to 26% across countries globally (56). HCV prevalence on the African continent is estimated to be 6.1%; SSA boasts the largest number of infections at 78 million (55, 90). Within SSA, Central Africa has the highest adult prevalence of chronic HCV at 9.7%, followed by West Africa with a prevalence of 8.3%. Within Central Africa, Burundi and Cameroon have the highest prevalence of 11.3% and 13.8%, respectively. Eastern and Southern SSA have chronic HCV prevalence of 5.5% and 3.8%, respectively (91). Prevalence of chronic HCV is lowest in North Africa at 2.8% (92). An estimated 71.1 million people worldwide are chronic HCV carriers, including approximately 18 million in Africa (76, 91). HCV prevalence increases with age, with the highest rate being reported amongst those >40 years. Chronic carriers are at risk of developing HCC with increasing age.

Like HBV, transmission of HCV is multi-modal. The most common route of transmission in SSA is parenteral. Although injection drug use is not as common in Africa as in many other parts of the world, it does present a major route of transmission. Historically an inadequately screened blood supply and reuse of needles in the medical setting also led to many infections (93, 94). In conjunction with high rates of sickle cell disease, blood transfusion has been a major route of acquisition (95). Vertical transmission is low, but more significant in the setting of coinfection with HIV. Prevalence of pediatric HCV varies from 0.05% to 0.36% in developed countries and from 1.8% to 5% in the developing world (96). Incidence of HCV vertical transmission has been shown to be 3% - 10% (97-99) and higher in infants born to mothers coinfected with HIV.

While multiple genotypes are present in SSA, genotypes 1 and 4 predominate. Prevalence of specific genotypes also varies by route of transmission and demographic characteristics (100). Treatment of HCV is guided by genotype and presence of cirrhosis. Thus, understanding genotype epidemiology is critical for planning of management programs in SSA. Although a vaccine for HCV is not yet approved, highly effective treatment has been available since 2014 when the first direct-acting antiviral (DAA) therapy ledipasvir/sofosbuvir was approved (101). Most of the DAAs available inhibit the HCV NS3/4A protease, NS5A protein, and NS5B polymerase. Prior to availability of DAAs, treatment with ribavirin and interferon-a was standard and had much lower cure rates. Provision of DAAs for treatment of HCV is critical for prevention of hepatocellular cancer and should be supported by national programs. Selection of DAAs for programs in SSA must be guided by local epidemiology.

### HCV-associated malignancies

Chronic HCV is a well-established risk factor for HCC, increasing the risk by 10–20 fold. HCV is the second major risk factor for HCC in Africa after HBV (102). HCC is the fourth most common cancer in Africa and has varying regional epidemiology (103). While HCV promotes hepatic (104, 105) and B-cell lymphoproliferative diseases (106, 107), it may not be the primary driver of tumorigenesis because it does not integrate into the host’s genome. HCV likely promotes tumorigenesis through repetitive damage, regeneration, and fibrosis seen during progression of cirrhosis (108). Annual incidence of HCC in persons with HCV-related cirrhosis ranges from 0.5–10% (109). The epidemiology of HCV disease progression to HCC in SSA has not yet been well-characterized but may follow trends seen elsewhere, which suggest a current increase in HCV associated HCC cases (109).

Risk factors for progression from chronic HCV to HCC have not been thoroughly characterized in the African population. Broader studies have shown an association with male sex, Hispanic ethnicity, HCV genotype 3, longer duration of infection, co-infection with HBV or HIV, markers of fibrosis, insulin resistance, obesity, diabetes, tobacco, and alcohol (51, 109) (110). HCV treatment with achievement of sustained virologic response (SVR) has the greatest impact on reducing progression (111). Several large studies of DAAs and meta-analyses (112) have demonstrated that HCC risk, while not eliminated, is reduced by 50–80% among persons who achieve SVR (113, 114). While HCC risk is reduced with SVR, the rates do not revert to baseline, especially among persons with cirrhosis. Long term follow-up of HCV infected patients who achieved SVR with DAAs has shown cumulative 1, 2, and 3-year risks of HCC of 1.1%, 1.9% and 2.8%, respectively. These incidence rates are at or below the threshold for cost-effective HCC surveillance (115).

### Future directions

HCV-associated HCC is a major public health problem in Africa. SSA health systems should anticipate an increasing number of HCC patients over the next several years. It remains unclear when the epidemic will peak, though its dynamics will likely trail that of HCV-associated HCC in more developed regions. National screening and prevention programs have been implemented and should be further expanded. International organizations should support local efforts through training and establishment of regionally sustainable facilities. Policy makers must prioritize control of viral hepatitis and its sequelae on the African continent.

The high prevalence of HCV infection in Africa necessitates augmentation of primary prevention efforts including vaccine development, as well as new approaches to reduce the burden of chronic liver disease. Vertical transmission of HCV in SSA must also be addressed. Mother-to-child transmission contributes to the SSA epidemic proportionally more than in other regions, where IDU tends to be the leading cause of infection. Standard screening of the blood supply must also be enforced.

While DAAs are highly effective for treatment of HCV infection, an estimated 95% of HCV infections remain undiagnosed worldwide (116, 117). Furthermore, prior HCV infection does not protect against HCV re-infection (118). Thus, development of a preventive HCV vaccine is essential to managing the epidemic; therapeutic vaccines to treat existing infection may also offer advantages of DAAs. Development of new assay methodologies and animal models facilitated discovery of partially effective antibody based vaccines in the eighties (119). Trials of viral vector vaccines were initiated in the nineties (120, 121). More recently studied vaccine platforms include HCV-like particles, recombinant proteins, DNA constructs, peptides, and novel viral vectors (122-124) (125) An effective HCV vaccine will likely need to induce robust neutralizing antibodies as well as multi-specific cellular immune responses. Advances in understanding of HCV pathogenesis in conjunction with innovative technologies have given rise to candidate vaccines currently in clinical trials (126). Deployment of an effective vaccine will be integral to HCV control in SSA.

Education programs targeting healthcare providers and high-risk populations should increase awareness about HCV infection, sequelae, prevention, and treatment. Societal knowledge expansion will be critical for engaging affected populations and breaking the cycle of disease transmission. Future research on interruption of vertical transmission, HCV vaccines and effective use of DAAs in the SSA context will be helpful. Table 2 shows key information on factors related to acquisition of infection, disease course, and control strategies for HCV.

## Epstein-Barr virus

### EBV genomic organization and protein products

Epstein-Barr virus (EBV) is a *Herpesviridae* whose tropism for lymphocytes has led to it being classified in the subfamily *Gammaherpesvirinae* (127). It was discovered in malignant B-lymphocyte proliferations of children’s jaws by Epstein and Barr in 1964 (128), consistent with its now established propensity for infection of the oropharynx and B cells (129). EBV is an approximately 172 kb double-stranded DNA virus composed of an icosahedral capsid surrounded by a cell membrane derived envelope into which viral glycoproteins are inserted. The genome encodes approximately 100 proteins that regulate gene expression, DNA replication, and immune responses; roles of several EBV proteins are still being characterized (130). The genome is divided by 0.5 kb terminal direct repeat (TRs) and internal repeat (IR) sequences into short and long sequence domains.

Several strains of EBV have been sequenced (131-134). However, open reading frames (ORFs), genes, and transcription or RNA processing sites are frequently referenced to specific *Bam*HI fragments, from A to Z in descending order of fragment size, because the first sequenced EBV strain B95-8 was sequenced from a cloned library of EBV DNA *Bam*HI fragments (135). Sequence analysis has been essential for characterizing EBV. This has enabled identification of four previously unrecognized ORFs whose functions are still being elucidated: BFRF1A is likely involved in DNA packaging by homology with other herpesviruses; BGLF3.5 may be an integumental protein; functions of BVLF1 and BDLF3.5 are less apparent (133).

EBV’s lifecycle starts with primary infection, then proceeds through latent and lytic phases. The virus enters a new host oropharyngeally and then infects naïve B lymphocyte by binding between the major viral envelope glycoprotein gp350/220 and the CD21 receptor (136) (137) Alternatively, CD35 can serve as receptor for EBV in the absence of CD21 (138). Viral glycoproteins gHgL and gp42 interact with HLA on the target cell surface to facilitate membrane fusion (138, 139). Infection of naïve B cells engenders polyclonal lymphoblastoid proliferation and expression of activation markers such as CD23, CD30, CD39 and CD70. The virus then enters “latency III” (cell proliferation), during which it expresses the antigens of the latency cycle: the 6 EBNAs (Epstein-Barr nuclear antigens): EBNA-1, EBNA-2, EBNA-3A, EBNA-3B, EBNA-3C and EBNA-LP; the 3 LMPs (latent membrane proteins): LMP-1, LMP-2A and LMP-2B; two non-polyadenylated non-coding RNAs, EBER1 and 2 (EBV-encoded RNA transcribed); and transcripts of the *Bam*H1 A region of the genome, BARTS (139, 140). Details of these protein functions have been published (139, 141-144). Of note, the LMP-1 region has been an attractive locus to characterize EBV’s biological, genetic, and epidemiological properties (145, 146). LMP-1 has been linked to biological changes that influence transmission, transformation, and tumor microenvironment (147).

As “latency III” progresses, B lymphocytes differentiate into memory B cells with somatic hypermutation of immunoglobulin (Ig) genes and form a germinal center. This marks the beginning of “latency II” (cell differentiation). When the B lymphocyte divides, episomal EBV will also replicate. It will then be in “latency I” and expressing EBNA1. A healthy immune system will control these lifecycle events such that the virus will enter “latency 0” during which B cells become long-lived resting CD27+ memory cells. Gene expression, with the possible exception of some EBNA1, ceases (139, 140, 148). EBV persists for life in B lymphocytes as a few copies of closed circular genomic DNA, or episomes, which duplicate with each mitosis due to EBNA1 (130, 140). It is estimated that 1 to 50 cells per million B cells are infected with EBV (140).

The lytic phase is divided into three phases by regulated gene expression: immediate-early, early, and late. Emergence from latency involves simultaneous expression of the immediate-early genes BZLF1 and BRLF1 (149-151). These genes encode the transactivating proteins ZEBRA (152) (also known as EB1 (153), Zta (154), and BZLF1) and RTA [also known as BRLF1 (155)], respectively. Both proteins can be expressed from a 2.9 kb R-Z RNA bicistron and a 3.8 kb minor R-Z RNA bicistron transcribed from the promoter R (Rp). ZEBRA is also expressed from a smaller 0.9 kb mRNA from the downstream Z promoter (Zp). In addition, another 0.9 KB message encoding putative fusion protein RAZ, which is composed of parts of RTA and ZEBRA, is transcribed at a low level (156). RAZ may be a ZEBRA inhibitor, though its role is still under investigation (157, 158). Synergy between ZEBRA and RTA suggests that low levels of both proteins can trigger the lytic cascade. The BRRF1 gene product has recently been shown to activate Zp and cooperate with RTA to induce lytic infection (159).Transcription of immediate-early genes does not require *de novo* synthesis, implying that physiological signals from the host may activate the lytic cycle (160). Early gene products include enzymes necessary for viral DNA replication. DNA amplification marks the beginning of the late phase, during which viral structural proteins are expressed and assembled into viral particles. DNA is packaged into these particles before release of infectious virions to infect new naïve B lymphocytes and new epithelial cells. Healthy asymptomatic people perpetuate the infection cycle by reactivating and excreting virus abundantly in their saliva (130, 140, 142).

### EBV prevalence and distribution in SSA

EBV is a globally ubiquitous virus. Spread of the virus through saliva means that exposure is nearly inevitable. Most people are infected at some point in their lives, often during childhood. Timing of primary infection is related to hygiene, socioeconomic and demographic factors. Infection is thought to occur later in more developed countries (161). Primary infection during early childhood is common in SSA, which is consistent with the role of EBV in childhood malignancy there.

Although most of the population is infected with EBV, manifestations vary across geographic regions. The high impact of EBV in SSA is reflected by its discovery in the region. In 1964, Denis Burkitt identified a tumor that was the most common childhood malignancy in equatorial Africa (162). The tumor, which subsequently became known as endemic Burkitt lymphoma, was found to harbor herpesvirus particles later recognized as EBV (128, 163). Endemic Burkitt is mainly found in areas of Africa and Papua New Guinea with increased malaria prevalence. Risk of progression to endemic Burkitt lymphoma may be related to severity of past malaria infection (164). The annual incidence of Burkitt lymphoma is 4-5/100,000 amongst children less than 18 years old from equatorial Africa, where 50% of tumors diagnosed during childhood and 90% of lymphoma cases are attributable to Burkitt lymphoma (165) (166).

As immunodeficiency related Burkitt lymphoma predominantly affects HIV-infected populations, it is most common where HIV prevalence is highest. Diffuse large B-cell lymphoma, for which up to 90% of cases in HIV-infected patients are associated with EBV infection, also follows HIV epidemiology (167). Such malignancies tend to occur in more advanced HIV. Given that SSA has the highest global prevalence of HIV and has been unable to control the HIV epidemic thus far, immunodeficiency related Burkitt lymphoma, diffuse large B-cell lymphoma (DLBCL), and other malignancies facilitated by immune-suppression will be more common in the region.

Of the 83,087 cases of Hodgkin lymphoma reported in 2020, 10,815 (13%) were in Africa. The region accounted for 4,315 (18.5%) of the 23,376 attributable deaths. Age-standardized incidence and mortality rates were close to the global average (168). A study using EBV-encoding RNA *in situ* hybridization found an EBV prevalence of 54% in Hodgkin lymphoma in Rwanda. Prevalence did vary across subtypes (169).

Geographic distribution of EBV subtypes is highly variable, as has been shown for other viral carcinogens, including HPV (170, 171) and HBV (27). These patterns are important for public health, biology, and diagnosis. They also reflect underlying immunological pressures that drive diversification through host-pathogen adaptations in disparate populations. Variation in EBNA-2 and EBNA-3 genes that underly classification of EBV into types 1 and 2 (133, 172) highlight geographic diversity, though Burkitt lymphoma and nasopharyngeal carcinoma (NPC) exhibit geographic distributions and age-specific patterns unexplained by simple EBV epidemiology (173-175). For example, NPC occurs with high incidence in Eastern and South-Eastern-Asia and in some areas of the Middle East and North Africa (176).

Lack of effective EBV infection control strategies and healthcare infrastructure requirements for effective management mean that SSA will continue to shoulder a significant burden of EBV associated malignancy. To further complicate matters, an individual can be infected with multiple EBV variants based on LMP-1 sequencing (177). The most practical approach for SSA may be to control risk factors, including HIV and malaria infection, that contribute to progression of EBV disease.

### EBV associated malignancies

Epstein–Barr virus (EBV) is a ubiquitous human lymphotropic herpesvirus with a well-established causal role in several cancers. It is associated with two general types of cancer: lymphoproliferative, which is mainly B-cell but may also affect T and NK cells; and epithelioproliferative, which is mostly nasopharyngeal but also includes certain gastric, mammary and pulmonary carcinomas (130). We will focus on malignant presentations in this paper, but one should also be aware of non-malignant presentations. For example, primary infection in healthy children <5 years old is typically asymptomatic, whereas 50% of adults and adolescents may develop infectious mononucleosis (IMN) (140, 178). Association of EBV with other conditions such as rheumatoid arthritis, multiple sclerosis, myasthenia gravis, and chronic fatigue syndrome remains under investigation (130).

Burkitt lymphoma is one of the most concerning EBV associated malignancies due to its rapid doubling time. It occurs when one of three translocations dysregulates the *c-myc* oncogene and is associated with proliferation of CD19^+^, CD20^+^, CD21^+^, CD10^+^, CD77^+^ and BCL6^+^ B cells whose origin is the germinal center (127). There are three types of Burkitt lymphoma: endemic, sporadic, and HIV related (140, 178). The endemic or “African” Burkitt preferentially affects bones of the face, most commonly the jaw. Endemic Burkitt lymphoma is most common in children 4 to 7 years old and is found in areas with high malaria endemicity (127). 90–95% of cases are EBV positive. Sporadic Burkitt affects young adults and is localized to the gastrointestinal or respiratory tract. It is associated with EBV in 15% of cases. HIV-related Burkitt localizes to the lymph nodes and bone marrow and it is associated with EBV in 30-40% of cases (179). Approximately 20% of lymphomas in PLWH are attributed to Burkitt and the risk of developing Burkitt has been estimated to be 200 to 1,000 times higher in PLWH compared to the general population (179). Treatment of Burkitt lymphoma is chemotherapy. Since EBV is in latency I in Burkitt lymphoma, expressing only EBNA1 and EBERs, adoptive immunotherapy is ineffective because CTLs do not recognize EBNA1 (180).

The role of EBV in Hodgkin’s disease had long been suspected based on increased incidence of Hodgkin’s in the setting of elevated anti-EBV antibodies, within 5 years of IMN, and in patients with IMN (179). The link was confirmed by identification of EBV DNA in Reed Sternberg cells (HRS). HRS are germinal center B cells that have undergone functional rearrangement of Ig genes but lack Ig transcription. Survival of HRS depends on presence of EBV (179). There are four histological types of Hodgkin’s lymphoma: nodular sclerosing, mixed cellularity, lymphocyte depleted and predominantly lymphocytic. Hodgkin’s lymphoma is associated with EBV in 40 to 65% of cases depending on histological type, age, and geography (181). Cells are in type II latency with expression of EBNA1, LMP1 and LMP2A proteins. The NF-KB pathway is activated due to presence of LMP1 in HRS or inactivation of NF-KB inhibitors when HRS cells are EBV-negative (140, 143, 178). Clinically, Hodgkin’s disease presents with fever, night sweats, asthenia, weight loss, lymphadenopathy, and hepatosplenomegaly.

Undifferentiated nasopharyngeal carcinoma (NPC) is an epithelial tumor responsible for approximately 50,000 deaths per year. The annual mortality rate in Southeast Asia and North Africa is 4 to 8/100,000 compared to 25/100,000 in Southern China where it is the leading cause of cancer regionally and amongst those who emigrate to other areas (127). Genetic and dietary factors (carcinogen ingestion) have been implicated in development of the disease in the setting of EBV infection. Malignant NPC epithelial cells uniformly contain the EBV genome. Peter Clifford demonstrated this association in Kenyan children in 1972 (182), though EBV was not classified as a group 1 carcinogen until 1997 (183). In NPC, the virus is in latency II, with expression of EBNA1 proteins and EBERs, and less consistently LMP1 and 2A (140, 143). MicroRNAs from the BART cluster are also found in plasma of patients with NPC. These microRNAs may play a role in the pathogenesis of NPC and have been proposed as a prognostic marker independent of EBV viral load (184).

Burkitt lymphoma, Hodgkin’s disease and nasopharyngeal carcinoma are examples of EBV associated malignancies seen in SSA. EBV can additionally cause several other malignancies. All have associations with specific phases of EBV latency. Diagnosis typically occurs after symptomatic clinical presentation; screening is not standard. Management may include radiation, chemotherapy, biologics, and immune reconstitution. EBV related malignancies present a significant burden across age groups in SSA.

### EBV future directions

High global prevalence of EBV in combination with elevated risk of progression to malignancy in SSA make EBV associated malignancies a public health priority for the region. Both prevention of infection and progression to malignancy need to be addressed. Unfortunately, exposure to EBV is almost unavoidable as it is transmitted by bodily fluids, particularly saliva. With no EBV vaccine currently available, most of the population will be infected. Effective strategies for preventing progression to malignancy are also lacking, though control of malaria could reduce incidence of endemic Burkitt lymphoma and control of the HIV epidemic could reduce incidence of immunosuppression associated Burkitt.

Further elucidation of the relationship between EBV-associated cancer and malaria will likely reveal prevention and mitigation opportunities. Epidemiologic associations have long been recognized, including high geographic correlation between Burkitt lymphoma incidence and intensity of *P. falciparum* transmission; correlation of Burkitt lymphoma incidence by age with age of acquisition of peak malaria immunoglobulin levels; decreased incidence of Burkitt lymphoma where malaria mortality has decreased; and older age of onset amongst people who migrated from low to high-intensity malaria areas compared to those born in high-intensity areas (185, 186). Such associations gave rise to discovery of EBV as a human oncogenic virus (187, 188) (189).

As EBV is not transmitted by mosquitoes, molecular mechanisms underlying the relationship between *P. falciparum* and EBV are of great interest. One study demonstrated that the cysteine-rich interdomain alpha region (CIDR1α) of *P. falciparum* membrane protein 1 acts as a polyclonal activator of B cells. CIDR1α increases B cell survival, preferentially activates the memory compartment where EBV persists, and induces virus production in latently infected primary B cells from healthy carriers and children with endemic Burkitt lymphoma (190). Studies have also shown that EBV can be activated by *P. falciparum via* cytidine deaminase-induced activation (AID) (191), which increases risk of DNA damage and lymphoma in murine models (192). This activation may facilitate preservation of B-cells with dysregulated c-myc expression that would normally undergo apoptosis (193). Thus, *P. falciparum* appears to be a risk factor for endemic Burkitt lymphoma through AID activation facilitating DNA damage and persistence of c-myc translocations. Additional research on mechanisms underlying the association between malaria and Burkitt lymphoma should be performed to identify disease intervention targets.

Research on the drivers of latency and its transitions in non-malaria endemic settings will also be useful. Analyses using recombinant forms of EBV may facilitate a better understanding of the role specific viral genes play in the EBV life cycle. Such information might lead to recognition of new vaccine targets (194) or therapeutic interventions targeting the function of essential EBV latent genes. Drugs that prevent binding of EBNA1 to oriP or interaction of FTTs with LMP1 could be developed (172, 195).

Development of an effective EBV vaccine should be prioritized. A vaccine that prevents infection would be ideal, though a vaccine that only reduces progression to malignancy would still have great impact. In conjunction with an improved understanding of mechanisms of EBV oncogenesis and development of an EBV vaccine, malaria vaccines currently being deployed and continuous improvement of the HIV prevention and care continuum could reduce associated Burkitt lymphoma cases in SSA. Fortunately, both malaria and HIV control efforts are supported through local and international programs.

Further understanding of mechanisms regulating EBV cell growth, survival and differentiation could also improve clinical management of those with EBV-associated disease. Adoptive transfer of EBV-specific CSF has proven useful in the treatment of immunoblastic B-cell lymphomas; this and other treatment strategies are currently being evaluated in patients with HD or NPC (196). Gene therapy strategies that exploit transcriptional regulation of the EBV genome or target the functional effects of latent EBV genes also offer possibilities for development of therapeutic and preventive strategies. Furthermore, continued refinement of longitudinal EBV viral load monitoring in transplant recipients presents opportunities for early intervention (194).

EBV genetic variation and host factors likely influence disease course. Further study on the mechanisms underlying epidemiologic associations may reveal risk modification strategies. Pathogenic differences in EBV strains may facilitate prioritization of vaccination targets. Genome deletions and polymorphisms may also be useful as predictive biomarkers.

SSA is highly affected by EBV related malignancies. Manifestations of EBV in the region are influenced by host factors and other regional conditions such as malaria and HIV. Inadequacy of healthcare infrastructure also means that cases may be missed or present with more advanced disease. Mitigating the socio-economic burden of EBV will require a combination of public health approaches, clinical research, and basic science investigation. Table 3 shows key information on factors related to acquisition of infection, EBV associated malignancies, and control strategies for EBV.

## Human papilloma virus

### Genomic organization and protein products

HPV is a small, non-enveloped virus. There are over 120 types that have been characterized by whole genome sequencing. These are divided into two major types: α (mucosal infectivity) and β (cutaneous). Not all types are oncogenic (197). Mucosal HPVs are split into “high risk” and “low risk” depending on if they cause potentially malignant or benign lesions. High risk types includes HPV 16 and 18, which cause ~70% of cervical cancer (198). Cutaneous HPVs, such as HPV 5, are associated with non-melanoma skin cancers.

All types of HPV have a ~8 kb double-stranded DNA genome. The genome is organized into early and late regions (including the long control region (LCR)). Most HPV genomes encode 8 proteins, 6 in the early region and 2 in the late region. The early proteins (E1, E2, E4, E5, E6 and E7) are highly expressed during the infectious or replicative cycle of HPV. E proteins are essential for genome replication, cytoskeleton remodeling, immune modulation, and modulation of the cell cycle. Two E proteins, E6 and E7, are oncogenic (199). However, E5 and E2/4/5 can also play a role in early oncogenic processes and an alternative carcinogenic pathway (200). Some HPV types have different isoforms of the E proteins that may play important roles in modulation of the host cell cycle, the viral lifecycle and cancer progression (201).The late proteins (L1 and L2) are essential for formation of the viral capsid and virus assembly.

Unlike many other viruses, there is no viremia or cell-death with HPV and most infections are asymptomatic; replication is exclusively intraepithelial and depends on the on the state of differentiation of the epithelial cell that the virus infects (202). Thus, most individuals may not know they are infected, which contributes to HPV being one of the most common sexually transmitted infections (203). Almost all adults have had at least one HPV infection; multiple types may integrate into the human genome (204). Given the asymptomatic nature of HPV infections, cancer may be the initial clinical presentation.

### HPV prevalence and distribution in SSA

SSA has a very high burden of HPV related cancers; 15.8% of all cancers for men and women can be attributed to HPV in the region (205). SSA is estimated to have the highest prevalence of cervical cancer rates in the world (age standardized incidence rate of 31.0 per 100,000 women) (206, 207). While HPVs are found both in men and women, women are disproportionally affected by HPV cancer (208). Data on HPV cancer trends in SSA is limited, with cervical cancer being best characterized. Incidence rates or HPV-associated oropharyngeal cancer, anal and penile cancer vary across the region; penile cancer rates are rising in some parts of Africa while the incidence of anal and oropharyngeal cancer seems to have remained low (209). Whether these data reflect actual incidence or changes in screening is unclear.

Throughout much of the region, there is a general lack of education about the risks of sexual activity and lack of resources for screening programs (such as cytology) or early treatment. For instance, in Mali, only 4.8% of women of reproductive age have been screened for HPV and cervical cancer. Lack of screening has resulted in a roughly 80% mortality rate for those diagnosed with cervical cancer (206). One study from the Democratic Republic of Congo showed that the highest risk group was women under the age of 30, particularly those who were HIV-positive (210). HIV-positive individuals in general have a higher risk for any type of HPV than HIV-negative individuals (211).

The distribution of HPVs is not uniform in SSA (208, 212). The high-risk HPV 16 is consistently found across SSA; predominance of other types varies regionally (212). Moreover, distribution may also be impacted by HIV status. In one study of women attending a clinic for cervical cancer screening in Sikasso, Mali, 63% of women were infected with high-risk HPVs (213). Interestingly, distribution of types differed by HIV status: HPV35/31/51-52-56 were associated with HIV-positive women while HPV31/56/52 were associated with HIV-negative women.

### HPV associated malignancies

It has been estimated that roughly 30% of infection-associated cancers are caused by HPV (214). Most HPV cells are cleared by the immune response. If the response fails, HPV infection becomes chronic, and the viral genome can replicate. As this occurs, early proteins E6 and E7 increase expression (199). E6 and E7 induce immunosuppression so infected cells are not cleared. Moreover, E6 and E7 are oncogenic proteins that result in genomic instability, disruption of the cell cycle, cell proliferation, immortalization, and malignant transformation (215).

Of the mucosal HPVs, 14 cause malignancies, with the majority being caused by HPV 16 and 18 (198). There are several different malignancies, the most common being cervical cancer in females. Females can also experience vulvar and vaginal cancer. Males can experience penile cancer. Both males and females can experience anal, oropharynx, and other head and neck cancers. It should be noted that some types of HPV only cause non-malignant warts, which will not be addressed in this topic on malignancies.

As cervical cancer is the fourth most common cancer amongst females globally and a leading cause of death (216), it is the-HPV associated malignancy that has garnered the most attention. HPV 16 is linked to approximately 50% of cases, while HPV18 is linked to another 20%. HPV 31, 33, 45, 52, and 58 are responsible for an additional 19% (217, 218). Additional HPV types are also classified as high-risk due to their role in cervical cancer, though they cause a much smaller proportion of disease (219). Additionally, men who have sex with men are at high-risk for anal infections and cancers; incidence of these cancer types has been increasing in this population (220) (221). PLWH also represent a unique population when it comes to HPV. PLWH clear the virus less efficiently than the general population and the overall incidence of HPV derived-invasive cancer is higher (222). They can also be infected with multiple HPV types (223). Further, in PLWH, the distribution of affected anatomical sites, HPV-types (224), and HPV derived cancers differs from that of the general population (225).

With increasing HPV vaccination coverage, the epidemiology of cervical cancer and other HPV associated malignancies may shift.

### Future directions

Currently, the best protection against high-risk cancer causing HPVs is vaccination (203). Widespread vaccine coverage, especially in areas where screening and early treatment programs are lacking could be an overall public health benefit and could lead to decreased incidence. Currently, it is estimated that only 20% of girls in SSA is vaccinated (226), which is one of the lowest rates in the world (227). Moreover, access to the nonavalent HPV vaccine is extremely limited, with the mono- and bivalent vaccines being most available. Furthermore, HPV related malignancy can be caused by HPV types not covered by the vaccines and HPV vaccination is not often prioritized as part of national vaccination programs in SSA (228). One study estimated that 90% vaccine coverage would reduce peak prevalence by 82% for Mali (229). HPV vaccines have evolved from bivalent products active only against HPV 16 and 18 to the current nonavalent vaccine. Current vaccines are effective against HPV 16 and 18 (206) and low-risk HPV 6 and 11 that cause the majority of anogenital warts. In addition to the aforementioned HPV types covered by the quadrivalent vaccine, the nonavalent vaccine also protects against HPV types 31, 33, 45, 52 and 58. The nonavalent product is indicated for prevention of cervical, vulvar, vaginal, anal, oropharyngeal and other head and neck cancers, genital warts, and some precancerous lesions caused by HPV (230).

While there are many different HPVs prevalent in the region, especially in Sub-Saharan Africa, the majority of cervical cancer is still caused by HPV 16 and 18, which means broader vaccination could have a major impact on HPV associated cancer and warts (211). Nonetheless, distribution of high-risk HPV types and associated malignancies could change with pressure from vaccines and societal factors. It is also likely to be different than the distribution seen in other geographic locations. Thus, ongoing regional characterization of HPV epidemiology will be needed to guide public health efforts.

Given the burden of cervical cancer in SSA, the WHO launched a campaign to increase vaccination coverage and eliminate cervical cancer (231). Following an educational program on HPV, it was found that vaccine acceptance was high in Bamako, Mali for both men and woman (206). However, disruptions to educational efforts and social mobilization have impacted such campaigns, resulting in disruptions to vaccine coverage, vaccine doses, and a downward trend in acceptability (231). It is important to reinvigorate such efforts. Combining these with related endeavors on primary and secondary prevention would further increase the impact (232). Additionally, secondary screening for cervical cancer could help decrease incidence (233). Secondary screening can be performed by visual assessment in resource limited settings such as SSA; when more resources are available, genotyping and cytology can be used to guide follow-up (234). The recommended frequency of screening, which varies with age and comorbidities, would be difficult to fully implement in the current SSA setting. Therefore, a multi-pronged approach is especially important given that HPV distribution is not uniform and current vaccines do not protect against all types of high-risk HPVs present in SSA (208, 212). Communication regarding the safety of vaccines is also essential.

Given the challenges to vaccination and that vaccination does not cover all types of HPV, there are many opportunities for research. For instance, we need to better understand how most HPVs are spontaneously cleared and to develop immunomodulatory vaccines or small molecule inhibitors that would be effective across all types (235). Additionally, vaccination is only effective prior to infection with HPV so development of immunomodulatory vaccines that could clear previous infections would be highly desirable. Table 4 shows key information on factors related to acquisition of infection, disease course, and prevention strategies for HPV.

## Human T-lymphotropic virus type 1

### HTLV-1 genomic organization and protein products

Human T-lymphotropic virus type 1 (HTLV-1), also called Human T-cell leukemia virus type 1, is a single stranded RNA retrovirus. Study of HTLV-1, the first oncogenic human retrovirus described, began in 1977 (236) (237) (238). Like HIV, the most common retrovirus, HTLV-1 targets T cells and reverse transcribes its RNA to make double stranded DNA that integrates into the host genome as a provirus. Unlike HIV, its primary mode for maintaining copy number during natural infection is clonal expansion as opposed to infection of new cells (239, 240). Analogous to HIV, the HTLV-1 genome contains *gag*, *pol* and *env* genes and is flanked by long terminal repeats (LTR) at the 5’ and 3’ ends. It has additional genes, particularly *tax* and *HBZ*, which regulate persistence and expansion of HTLV-1 infected cells. Activity varies depending on the balance of sense and anti-sense transcription (241). At this time, genomic variation is not fully characterized and association of mutations with specific clinical phenotypes is unclear (242). While pathogenic mechanisms of HTLV-1 remain under investigation, induction of cellular proteins that induce transformation plays a role; pure insertional mutagenesis or capture of a cellular proto-oncogene are unlikely (243).

Tax is a transcriptional activator/repressor capable of modulating expression of multiple cellular genes. It also interacts directly with diverse proteins, is antiapoptotic and promotes cell proliferation (244, 245). The tax protein can immortalize cells *in vitro* and forced expression in transgenic mice leads to development of leukemia/lymphoma (246, 247). The primary mechanism of tax transformation is related to cell cycle reprogramming and inhibition of DNA repair (248). Tax also induces NFκB activity, which stimulates expression of cytokines and their receptors, including those of IL-13, IL-15, IL-2, IL-2Rα and co-stimulatory surface receptors (OX40/OX40L) (249-251). This activity mimics the chronic inflammatory process, which promotes oncogenic progression of many cancers, and triggers proliferation of T-cells to amplify the pool of HTLV-1 infected cells. Unlike other cancers in which the inflammatory process is mediated by immune cells in response to an oncogenic insult, HTLV-1 progression is directly induced by Tax. In addition to NFkB promoters, Tax also regulates expression of cellular transcriptional promoters through interaction with cyclic response element binding protein-AMP (CREB) and serum response factor (SRF) (252).

*HBZ* is located on the minus strand of the HTLV-1 provirus. It encodes a Basic Leucine Zipper Domain (bZIP) protein called HTLV-1 bZIP factor (HBZ). HBZ regulates 5’LTR transcription and modulates several cellular signaling pathways involved in cell growth, immunologic responses, and T-cell differentiation. It has been known to promote leukemic cell proliferation and induce T-cell lymphoma and systemic inflammation (253).

Compared to other retroviruses, HTLV-1 is genetically conserved and evolves slowly *via* accumulation of point mutations and recombination (254). Molecular studies suggest an evolutionary rate between 5.6 x10^−7^ and 1.5 x 10^−6^ substitutions/site/year. This slow rate has been attributed to persistence by clonal expansion as opposed to new infectious cycles using reverse transcriptase (255).

Phylogenetic analyses of the LTR region have demonstrated several HTLV-1 genotypes and subgroups which reflect geographic distribution (256-259). The three major molecular genotypes are: Cosmopolitan a-genotype, Central African b-genotype, and Australo-Melanesian c-genotype. The minor genotypes d, e, f, and g have also been characterized in Central Africa (260, 261), particularly in local pygmies (257, 262, 263). The Cosmopolitan genotype HTLV-1a can be subdivided into geographically related subgroups: Transcontinental (a-TC), Japanese (a-Jpn), West-African (a-WA), North-African (a-NA), and Senegalese (a-Sen). Genetic diversity is low within subgroups. Genotype HTLV-1b is found in Central Africa, and is the major genotype in Gabon, Cameroon, and Democratic Republic of Congo. Strains from the HTLV-1d genotype constitute a few percent of strains in Central African countries; genotypes e, f, and g have been sporadically reported in Cameroon, Gabon, and the Central African Republic. HTLV-1c, the most divergent genotype, is only in Australo-Melanesia (255).

### HTLV-1 prevalence and distribution in SSA

WHO estimates that 5-10 million people worldwide are infected with HTLV-1 (238), though this is probably an underestimate due to inadequate data (264, 265). While HTLV-1 has spread globally, its geographical distribution is not uniform. The virus is endemic in Africa (266, 267) and is frequently found in Melanesia, Papua New Guinea (268), Solomon Islands and Australian aborigines (264). The greatest genetic diversity is observed in SSA, where six of the seven genotypes have been found; five of these are mainly in Central Africa (261).

Studies representing a total of 42,297 participants have shown seroprevalences of 4.16% (95% CI 2.43-6.31%), 2.66% (95% CI 1.80-3.68%) and 1.56% (95% CI 0.48-3.15%) in Central, West and Southern Africa, respectively. Of note, prevalence varied significantly by year (269, 270). In terms of specific populations, seropositivity rates range from 1 to >5% of West African blood donors and pregnant women in Ghana (271), Benin (272, 273), Mali (274) and Guinea-Bissau (275). One serologic survey in Guinea-Bissau in 2000 showed a prevalence of 9.3% amongst blood donors and 3.6% in the general population (276); a prior survey showed a prevalence of 3.3% of pregnant women (264). Although serologic data has been the foundation for prevalence estimates, it may be confounded by antibody cross-reactivity with malaria antigens (277-279).

HTLV-1 is transmitted primarily through infected body fluids, including blood, breast milk and semen. Risk factors include unprotected sex, injection drug use, and transplantation of tissue, blood, and blood products (280-282). Transfusion of HTLV-infected blood is the most effective mode of transmission due to presence of infected lymphocytes. Estimated seroconversion rates range from 27% to 63% after exposure to seropositive cellular blood components. Fortunately testing of blood donations for HTLV-1 in several high-income countries has greatly reduced transfusion-transmitted HTLV-1 (283). Expansion of blood product screening, particularly in SSA, presents an opportunity for improving control of HTLV-1 and its sequelae.

Interruption of mother-to-child transmission is also an intervention target. The estimated mother-to-child transmission rate is 18-30% (284, 285), which occurs primarily through breast-feeding as opposed to before or during birth. Breastfeeding longer than 6 months has been associated with transmission, leading to the hypothesis that shortening the duration of breastfeeding may reduce HTLV-I transmission. However, vertical transmission is more likely multi-factorial as approximately 3% of children who were not breastfed still became infected in intervention studies (286-289). As breast-feeding is nearly ubiquitous in SSA, control of vertical transmission will be extremely difficult in the region.

Of HTLV-1 infected patients, an estimated 6.6% of males and 2.1% of females develop adult T-cell leukemia/lymphoma (ATL) (264). There is also a potential association of both HTLV-1 infection and its sequelae with familial clusters, with up to seven people in a family affected (290, 291). ATL and HTLV-1-associated myelopathy/tropical spastic paraparesis (HAM/TSP) were the most frequently reported sequelae within families. Clustering may be related to familial transmission of HTLV-1, though other factors may be involved (292). Characteristics of gender and family dynamics may hold clues to HTLV-1 associated malignancy in Sub-Saharan.

### HTLV-1-associated malignancy

Approximately 10% of HTLV-1 infected individuals develop associated disease; most people remain asymptomatic (261, 293). The virus is best known for causing ATL and HAM/TSP, though it is also associated with infectious dermatitis, uveitis, crusted scabies, inflammatory rheumatoid conditions, peripheral neuropathies, myositis, and atherosclerosis (264, 294, 295). This section will focus on ATL because it is the only malignancy associated with HTLV-1.

ATL was the first identified HTLV-1 associated disease, hence the virus’ name. It is a form of leukemia/lymphoma that derives from clonal expansion of infected CD4 T cells. These CD4 cells are presumably part of a long-lived pool of memory T cells with stem cell properties, though tumors express other T-cell associated antigens (296). ATL cases do not have distinct molecular or karyotypic abnormalities. Nonetheless, all cases have clonally integrated HTLV-1, with the most clinically aggressive variants manifesting the most extensive chromosomal alterations (297). Peripheral blood cells may have distinct hyperlobulated nuclei (298). ATL is classified into four clinical subtypes: acute (60%), lymphomatous (20%), chronic (10%) and smoldering (10%); primary cutaneous is an additional provisional subtype. Bone marrow infiltration, lymphadenopathy, skin involvement, hypercalcemia and immunosuppression occur to varying degrees (299, 300). Progression from chronic and smoldering forms to acute disease can occur (301). Diagnosis is based on immunophenotype, cell morphology, clinical features and presence of HTLV-1 (302).

Treatment of ATL depends upon clinical subtype. Intervention may be deferred for non-unfavorable chronic and smoldering subtypes, thought patients should remain under surveillance. Acute, lymphomatous, and unfavorable chronic ATL should undergo chemotherapy. Patients are at risk for hypercalcemia, tumor lysis syndrome and opportunistic infections during treatment. Allogeneic hematopoietic cell transplantation can be considered for those with an appropriate donor; this strategy may engender a beneficial graft-versus-leukemia effect (303). Regardless of treatment, prognosis is guarded.

Regions with highest prevalence of HTLV-1 infection have higher incidence of ATL. This includes Japan, intertropical Africa, the Caribbean, Central and South America, Romania, and northern Iran (304). Japan has the highest regional incidence, with annual rates approaching 60/100,000 HTLV-1 carriers and an estimated lifetime risk of 6-7% amongst men and 2-3% amongst women (305). Most cases stem from HTLV-1 contracted by breast-feeding and during early childhood (306). It has been estimated that the risk of ATL in perinatally infected people approaches 25% and that 2-4% of people will develop ATL within 30 years of HTLV-1 infection (307).

Although not a malignancy, the HTLV-1 associated condition HAM/TSP merits mention here. This is a chronic and progressive condition of adults in equatorial areas. It causes weakness, muscle stiffness and spasms, sensory disturbances, and sphincter dysfunction. The lifetime risk of HAM/TSP in HTLV-1 carriers is approximately 0.25-3%, which is lower than the risk of developing ATL (308, 309). There is no specific treatment, and the condition is associated with high morbidity and mortality (310, 311). As noted above, HTLV-1 is also associated with several other non-malignant manifestations.

### Future directions

There is currently no effective and scalable prevention or cure for HTLV-1 infection. Asymptomatic infection is not treated, and management of associated disease is suboptimal. Transmission can occur from asymptomatic carriers through contact and organ or blood donation. The severity of HTLV-1-associated diseases and limited treatment options highlight the need for preventative vaccines and new therapeutic interventions (312). Unfortunately, no HTLV-1 vaccine candidate has yet progressed to efficacy studies in humans. Screening of blood and organ donations is helpful but does not address other major modes of transmission; parenteral, sexual, and vertical transmission must also be interrupted. Public health entities should support community-based strategies to increase awareness and reduce viral transmission.

While research on prevention of HTLV-1 infection and management of associated diseases is ongoing, clinicians can focus on early detection, particularly of ATL and HAM/TSP. This will be a challenge in SSA, where HTLV-1 infection is endemic and healthcare resources are severely limited. As with many medical conditions in SSA, people often rely on scarce public health resources which they access infrequently. Consequently, cases commonly present in advanced stages. Strategies for risk prediction should thus be developed. While proviral load has been suggested as a potential indicator, no single biological correlate of progression has been identified. In addition to proviral load, age, family history of ATL and HTLV-1 testing were identified as risk factors for development of ATL in a prospective study in Japan (313). Additional investigation can facilitate optimization of screening approaches.

In addition to research on prevention and management strategies, further epidemiologic characterization should be conducted. Understanding of subtype distribution, transmission patterns and factors that influence risk of progression will enable targeting of resources where impact will be greatest. A multipronged approach will provide the best prospect for SSA to mitigate HTLV-1 infection and its consequences. Table 5 shows key information on factors related to acquisition of infection, the associated malignancy, and control strategies for HTLV-1.

## Human herpesvirus-8 or Kaposi sarcoma-associated herpesvirus

### HHV-8 genomic organization and protein products

HHV-8 is a double-stranded DNA virus of approximately 140 kilobases. Its genome is structured like other herpesviruses, with a single long unique region flanked by GC-rich terminal repeats at both the 3’ and 5’ ends (314). While the central region of the genome is highly conserved, both ends demonstrate significant variability. Sequencing has revealed 86 genes, 22 of which encode immunomodulatory proteins (315). ORF-K1 at the 5’ end encodes a glycosylated transmembrane protein with roles in signal transduction, viral reactivation, endothelial cell immortalization, and host immunity (316, 317). High variability of the ORF-K1 distinguishes the six main HHV-8 clades, which are A, B, C, D, E and F (317-319). The K15 gene at the 3’ end encodes and integral membrane protein and gives rise to the P (predominant), M (minor), and N genotypes. Nine additional loci with less variability than K1 and K15 can be used for subtype characterization (320, 321). Although the HHV-8 gene map has changed little since it was first characterized in 1996 (322), additional evaluation of HHV-8 genotype nomenclature based on evolving methodology is underway (323).

Consistent with other herpesviruses, the HHV-8 life cycle includes lytic and latent phases. Latency is the default pathway; it has been seen in nearly all tissues (324, 325). During this phase, the viral DNA constitutes a non-integrated plasmid and has limited expression. One critically expressed gene used for diagnosis of KS and Primary Effusion Lymphoma (PEL) is latency-associated nuclear antigen (LANA) from ORF73 (326-328). LANA can repress transcription of lytic genes and recruit other transcriptional repressors (329). Lifelong latency is established primarily in B lymphocytes that reside in tissues, with genomes being difficult to detect in plasma or PBMC (330).

Reactivation of HHV-8 from latency is initiated when expression of the replication and transcription activator (RTA) protein is stimulated. RTA is known as the master lytic switch protein (331). It competes with LANA for binding to suppressive co-factors, leading to transactivation of 34 lytic genes (332). Transactivation of the RTA promoter creates a feed-forward loop to overcome repression and further increase lytic gene expression (333). Environmental stimuli such as hypoxia and calcium signaling can promote RTA activation, which may influence anatomic localization of associated lesions (334, 335).

Entry of HHV-8 to the lytic phase engenders oncogenesis. Thus, addressing HHV-8 infection and associated disease is essential for prevention of morbidity and mortality.

### HHV-8 prevalence and distribution in SSA

While HHV-8 causes significant morbidity and mortality, most infections remain latent. Thus, prevalence is much higher than reflected by cases of associated malignancy. Estimates based on seroprevalence in adult populations in Africa or of African origin suggest that 36-90% of people have been infected. Some studies showed increasing seroprevalence with age (336-339). In Gabon, where seroprevalence based on plasma anti-LANA antibodies was only 36%, locality, age, sex and ethnic group were not correlated (340). The generally high prevalence of HHV-8 infection in SSA means that a large proportion of the population is at risk of HHV-8 associated disease.

Within SSA, HHV-8 genotypes A and B predominate, along with a lower proportion of F (341). Within genotype A, clade A5 is most prevalent (342). Phylogenetic analyses indicate that circulating strains are dynamic, with those circulating today being distinct from those circulating at the height of the HIV epidemic. However, unavailability of banked samples makes it difficult to elucidate temporal evolution in Africa (314).

As HHV-8 genotype influences risk of disease progression, it is important to understand the regional epidemiology of underlying infection. Analysis of ORF K1-VR1 sequences in blood, serum, and saliva of 38 patients with Kaposi Sarcoma (KS) found that genotype A was associated with rapid progression (12/17 cases) and that C was most common in slow progressors (6/7 cases). Genotype A was also associated with higher viral load in the blood (343). A South African study that performed ORF-K1 subtyping of tissue biopsies from 86 KS patients, 81 of whom had AIDS and 5 of whom had African endemic-KS, showed that subtypes A5 (38 AIDS + 4 African endemic/86) and B2 (16 AIDS/86) predominate. A5 and B were found in African blacks and individuals of mixed ancestry. 62/86 (72%) had >10 lesions at presentation; A5 was associated with >10 KS lesions in patients with AIDS. Thus HHV-8 subtypes A5 may be associated with more extensive disease (318).

Genomic polymorphisms impact disease presentation and may vary by anatomic site. Whole genome sequencing of Zambian KSHV revealed phylogenetic segregation from Western sequences, including polymorphisms in the more conserved genes. Such geographic diversity may engender differences in pathogenesis (319). Conversely, analysis of KSHV genomes from patients and controls in Cameroon revealed that recombination is common, suggesting multiple KSHV infections can occur. Yet sequence variation did not correlate with disease risk (344). A Ugandan study of KSHV genomes from tumors and oral swabs found that genomes were identical at the point mutation level within individuals but intra-host tumor-associated KSHV mutations and genome sequences impacting protein coding were present (345). On the host side, an association likely exists between HLA polymorphisms and susceptibility to KSHV infection and related diseases (346).

Given the high seroprevalence of HHV-8 subtypes associated with more severe disease in SSA, prevention, screening, and management strategies are critical. Saliva serves as the main mode of transmission and infection is common within families. Mother to child transmission and sex also contribute to varying degrees in different populations (347). Screening for HHV-8 is not standard, with infection typically being evaluated only after someone presents with a disease manifestation. Management varies from reconstitution of the immune system to multiple cycles of chemotherapy. Public health strategies to address HHV-8 in SSA must balance the challenge of prevention, cost of disease management and socioeconomic context.

### HHV-8 associated malignancies

Major malignancies associated with HHV-8 include KS, PEL and Multicentric Castleman’s Disease (MCD) (348). HHV-8 positive diffuse large B-cell lymphoma and germinotropic lymphoproliferative disorder also occur. These cancers are usually found in immunodeficient patients, but can affect immunocompetent individuals (349) Manifestations typically arise many years after acquisition of infection and only in a subset of those who are infected.

Primary HHV-8 infection is usually asymptomatic (350), with most people seroconverting without progression to malignancy. However, manifestations vary amongst populations by age and risk factors (351, 352). Immunocompromised people can develop more generalized manifestations as well as rapid onset KS (353).

KS is the most common and well-studied malignant manifestation of HHV-8 infection. It remains one of the most frequent cancers amongst HIV-infected patients (354) and children from endemic regions, including Central, Eastern, and Southern Africa (355). There were 34,270 new cases of KS and 15,086 associated deaths reported worldwide in 2020. Actual morbidity and mortality are probably greater. Africa shouldered the greatest burden, with 25,010 (73%) of the cases; 15,457 of those were in the Eastern Africa region. 13,066 (87%) of the deaths occurred in Africa, with 9,121 of them in the Eastern Africa region (356).

KS is an angioproliferative, cytokine-driven neoplasm which frequently presents with peripheral skin lesions (357). Lesions also affect the mucosal membranes and viscera, where manifestations may be due to mass effect or obstruction. Pediatric KS in endemic regions is known to present in lymph nodes and without skin findings (358, 359). Four types of KS are recognized. Classic KS is a slowly progressive cutaneous disease mainly seen in elderly Mediterranean and Jewish males (360). Endemic KS affects all ages and is particularly common in SSA (361). This form is not immunosuppression-associated and is more aggressive, disseminating to lymph nodes, bone and skin (362). Solid organ transplant-associated KS occurs post-transplant and may be due to HHV-8 transmission during transplant. Disease duration is usually related to immunosuppression, with regression occurring when the immune system reconstitutes (363). Epidemic KS, also known as AIDS-related KS, is >20,000 times more common in AIDS patients compared to the general population. It can also occur in other immunosuppressed hosts, but is still much more common in AIDS patients (364).

KS-immune reconstitution inflammatory syndrome (KS-IRIS) can occur when the immune system of an immunosuppressed patient with KS recovers. Symptoms such as increase in lesions, fever, inflammation, and pain occur when the immune system suddenly recognizes antigens to which it was previously not responding. KS-IRIS is typically seen in advanced HIV patients who initiate ART and had KS, though lesions may have been subclinical (365). It is associated with significant morbidity and mortality, particularly in Africa where adjunctive therapies may be less available (366). A study of 58 KS IRIS patients in SSA and the UK found that KS associated mortality was 3.3 fold higher in SSA compared to the UK and was predicted by KS-IRIS, lack of chemotherapy, pre-ART CD4 <200 cells/μL, and detectable baseline KSHV DNA. All 19 study deaths occurred in the SSA cohort (367).

KS-Associated Herpesvirus Inflammatory Cytokine Syndrome (KICS) is a consequence of HHV-8 seen in HIV+ patients with elevated plasma HHV-8 viral loads. It entails systemic inflammatory manifestations and high levels of IL-6 and IL-10 (368, 369). KICS overlaps somewhat with other HHV-8 associated conditions and has high mortality rate.

MCD is an uncommon lymphoproliferative disorder that can present with generalized lymphadenopathy, hepatosplenomegaly, and constitutional symptoms including fever, night sweats, weight loss, and fatigue. Patients are typically cytopenic and can have hypoalbuminemia, polyclonal hypergammaglobulinemia and elevated inflammatory markers. MCD often occurs in the setting of HIV or other etiologies of immunosuppression. It involves multiple areas of lymphadenopathy that is histopathologically on the spectrum of hyaline vascular to plasmacytic (370, 371). HHV-8 causes approximately half of the MCD cases (372). It should be noted that the unicentric form of Castelman’s Disease, which affects a single anatomic region, is not associated with HHV-8 infection.

Although specific diagnostic criteria are not available, MCD is usually identified through histopathologic examination of lymph node biopsy. Fluorodeoxyglucose positron emission tomography (FDG-PET) combined with computed tomography (CT) will reveal activity at multiple sites, though activity is lower than seen with aggressive lymphomas (373). This diagnostic approach is not broadly feasible in resource limited settings. It is likely that many cases of MCD go unrecognized in SSA. Management of MCD depends on the presence of concomitant KS, extent of organ involvement and underlying immunosuppression. Patients with HIV will use ART. Rituximab can be used for simple MCD without organ failure or KS. Chemotherapy is used in more extensive cases. 5-year survival is >90% with proper management, though patients remain at risk for developing non-Hodgkin lymphoma (374).

MCD in the African context has unique features and management challenges. Characterization of MCD in SSA has been limited due to incomplete case recognition. Underdiagnosis of MCD in SSA was suggested by immunohistochemistry positive LANA and viral IL-6 in 3/64 reactive appearing lymph nodes from a Ugandan study (375) and identification of only 35 cases in South Africa from 1990-2014 (376). The first case of MCD in Malawi was reported in 2014 (377). The same group subsequently reported on a Malawian cohort of MCD, lymph node KS and NHL. MCD was typically diagnosed late and associated with high mortality (378). Building on this study, the first well-characterized prospective cohort of MCD in the context of HIV SSA revealed only HIV+ KSHV+ MCD, which differs from the heterogenous epidemiology of MCD in high-income settings where cases can be HIV− and KSHV−. This is not surprising given the high regional prevalence of both HIV and KSHV (376).

PEL is a rare and aggressive B-cell lymphoma that typically occurs in HIV-infected patients, though it can occur in other immunosuppressive conditions (379, 380). Tumor growth depends on HHV-8 infection, and sometimes co-infection with EBV (381, 382). In addition to containing HHV-8 genetic material, the malignant monoclonal B cells express surface CD38 (383). PEL tends to occur on serosal surfaces in body cavities such as the peritoneal, pleural, and pericardial spaces, which is why it is also known as body cavity based lymphoma. Clinical presentation is usually due to fluid accumulation, though presentation with solid tumors is possible (384). Treatment is chemotherapy with ART and prognosis is generally poor, with survival being approximately 3-6 months (385). The burden of PEL in SSA remains unclear, as cases are likely much more frequent than recognized in this endemic area (386, 387).

### Future directions

SSA’s confluence of high HHV-8 infection prevalence, high proportion of more aggressive HHV-8 genotypes, burden of HIV and lack of healthcare infrastructure have made HHV-8 associated malignancy a major cause of morbidity and mortality in the region. While KS, the most common malignant manifestation of HHV-8, may be recognized clinically, less common manifestations such as PEL and MCD are inevitably underdiagnosed. Thus, diagnosis and treatment strategies must be developed within the context of SSA. For example, rapid case ascertainment, the expeditious evaluation of a potentially rapidly fatal condition shortly after diagnosis, is feasible for investigation KS in East Africa but is mostly used in resource rich settings (388). One study using rapid case ascertainment of newly diagnosed KS amongst PLWH in Kenya and Uganda showed that most patients had advanced disease despite recommendations for ART for all PLWH. Advanced disease was heterogeneous and raised questions about the possible need for different treatment strategies in this group (389). Also, the most effective chemotherapeutic agents are rarely used in SSA due to high cost. Yet, modeling can reveal the impact of therapies on life expectancy and their cost effectiveness to inform efficient allocation of resources. Given the high prevalence of HIV in SSA, modeling for AIDS-associated KS is particularly salient (390). In the same context, T-cell sparing treatments of KSHV associated diseases should be considered (391). Evaluation of treatment outcomes with respect to epidemiology of KSHV associated conditions in children and adolescents should also be enhanced. A study in Southwestern Tanzania showed favorable outcomes in a varied cohort, but generalizability of these findings is unclear (392).

As appropriate management requires evaluations of the malignancy and host factors that may not be feasible given available resources, prevention of HHV-8 infection would seem to be a desirable goal. Minimization of progression to malignancy would be another key target. Effective HHV-8 infection prevention strategies remain unclear, particularly because routes of transmission have not been fully elucidated. Saliva is the main route, though transmission through sex, blood and organ transplant also occur. Transmission *via* saliva in combination with high underlying prevalence in SSA make exposure likely in the general population. Furthermore, there is no vaccine for HHV-8.

Once someone is infected with HHV-8, likelihood of progression to malignancy depends on host factors. The main risk is immunosuppression, most often associated with HIV infection. SSA has the greatest worldwide burden of HIV and sub-optimal, though improving, rates of identification and effective management of PLWH. Thus, prevention of progression to malignancy will largely hinge upon controlling the HIV epidemic. Fortunately, progress is being made in this area, including expansion of public health programs with international support.

Considering the limited options for prevention of HHV-8 infection and progression to malignancy in those who are infected, research should be expanded in these areas. Development of an effective HHV-8 vaccine should be prioritized. The vaccine would ideally prevent infection by all genotypes but would still be helpful if it just covered the high-risk genotypes prevalent in SSA. It should induce sterilizing antibody responses to prevent primary infection along with T-cell responses to inhibit cancer development in those who get infected (393). Even a vaccine that does not completely prevent infection, but prevents complications, would be valuable. Platforms used in the recent rapid development of vaccines for COVID-19, and other infections may facilitate development of an HHV-8 vaccine. Other areas of research could include scalable strategies for diagnosing less common HHV-8 associated malignancies, treatment for resource limited settings, and transmission prevention techniques. These would be enhanced by further characterization of HHV-8 epidemiology, continued investigation of pathogenesis, understanding of host factors including genetic and epigenetic contributors, and environmental modifiers.

HHV-8 is a common infection in SSA, as well as a significant cause of morbidity and mortality. Much work remains to be done on prevention of infection, treatment of complications and novel research to improve control. Table 6 shows key information on factors related to acquisition of infection, associated malignancies, and control strategies for HHV-8.

## Human immunodeficiency virus

### HIV genomic organization and protein products

While HIV-1 and HIV-2 are genetically distinct, their genome, proteins and end stage disease have many similarities. In this section, HIV refers to both HIV-1 and HIV-2; discussion relevant to only one type of HIV will be specified accordingly.

HIV is an RNA virus with an approximately 9kb genome containing nine genes and encoding fifteen proteins. *gag, pol* and *env* are the three major genes constituting most of the genome. *gag* encodes the structural proteins Matrix, Capsid, Nucleocapsid, and p6; *pol* encodes the enzymes Protease, Reverse transcriptase, and Integrase; *env* encodes envelope proteins gp120 and gp41. The smaller remaining genes code for regulatory proteins Tat and Rev, as well as accessory proteins Vif, Vpr, Vpu/Vpx, and Nef (394, 395). Infected cells have integrated DNA copies of the virus, or proviruses, which are non-defective or defective. Viruses produced from non-defective proviruses and proteins produced from defective proviruses can facilitate oncogenesis.

Treatment with antiretroviral therapy (ART) targets specific HIV proteins at key steps in the viral lifecycle, e.g., reverse transcriptase inhibitors, protease inhibitors, and integrase inhibitors. Both HIV-1 and HIV-2 are treated similarly with ART. However, the non-nucleoside reverse transcriptase inhibitors (NNRTI) cannot be used in HIV-2 due to structural differences in the NNRTI binding pocket of HIV-2 reverse transcriptase (396). Although three HIV-1 cases have achieved sterilizing cure through stem cell transplant, lifelong ART is generally required because there is no scalable HIV cure. If ART is stopped, the virus rebounds from the integrated proviral DNA reservoir (397). While ART has toxicities that engender concern about promotion of oncogenesis, it inhibits the oncogenic effects of HIV by reducing production of oncogenic proteins and controlling viral load.

### HIV prevalence and distribution in SSA

Of the estimated 37.7 million people living with HIV (PLWH) globally in 2020, approximately 25.3 million were in SSA. Most of the infections are HIV-1, with HIV-2 accounting for an estimated 1-2 million cases (398). Eastern and Southern Africa shouldered the greatest burden with 20.6 million PLWH compared to 4.7 million in Western and Central Africa. Access to ART was around 78% for the general adult population, but lower for pregnant women in Western and Central Africa and higher for pregnant women in Eastern and Southern Africa. The only UNAIDS region with greater adult access to ART is Western and Central Europe and North America, where 83% of adult PLWH have ART access. Women and girls accounted for 63% of new HIV infections in SSA during 2020. Amongst adolescents 15-19 years old, approximately 85% of new infections were in females (399). Evidence suggests substantial variation in HIV prevalence across SSA localities. A high-resolution study of adult epidemiology showed generally higher prevalence in southern regions, with pockets of high prevalence in other areas. Risk factors did not consistently align with prevalence; condom use was higher in southern areas and male circumcision was higher in northern and central regions (400).

While HIV prevalence is decreasing in SSA, most infections continue to occur amongst females. Heterosexual sex is the primary mode of transmission. Populations disproportionately affected include sex workers, men who have sex with men (MSM), injection drug users (IDU), transgender persons and prisoners (401). Prevalence is particularly high in areas of high economic activity, likely related to seasonal work and commercial sex. High prevalence areas are clustered around high traffic regions including border crossings, major highways, ports, and areas of migration (402). SSA’s already high HIV prevalence is exacerbated by the local social, political, and economic climate. High levels of poverty, lower levels of education, and poor healthcare infrastructure hinder achievement of the UNAIDS “95-95-95” HIV cascade of care targets, under which 95% of PLWH know their status, 95% of those diagnosed are on ART and 95% of those on ART achieve viral suppression by 2030 (403).

Young adults have especially inadequate access to sexual health resources. Differences between male and female sexual debut, age disparate sex, transactional sex, multiple partnerships, low condom use and high background STI rates contribute to vulnerability, particularly amongst young women (404). While HIV pre-exposure prophylaxis (PrEP) consisting of oral ARV taken regularly or around sexual activity is approved, uptake in SSA has been inadequate. Challenges including target population engagement, use associated stigma, adherence requirements, healthcare infrastructure, provider unfamiliarity, community awareness and accessibility highlight opportunities for national public health program development (405, 406). Recent approval of injectable cabotegravir as PrEP may alleviate some barriers (407).

While HIV-1 and HIV-2 share transmission routes and cause AIDS, it should be noted that HIV-2 has lower rates of transmission and slower clinical progression. HIV-2 is also more geographically localized than HIV-1. Most people with HIV-2 are in West Africa, or countries with strong ties to the region such as France, Spain, and Portugal (398). Furthermore, HIV-2 prevalence in West Africa seems to be declining, likely due to lower virulence and transmissibility (408). The lower prevalence and pathogenicity of HIV-2 contribute to it being less well studied and underly the focus of HIV control efforts on HIV-1.

Understanding the HIV epidemic in SSA, and control strategies, will be key to minimizing HIV associated complications. PLWH are at increased risk for morbidity and mortality, including development of malignancy. Prevention of HIV and associated cancers will reduce socio-economic burden on the region.

### HIV-associated malignancies

HIV is well recognized for enabling oncogenic potential of other viruses through decimation of immunologic function that typically suppresses oncogenic activity. While both HIV-1 and HIV-2 are endemic to SSA, they carry different risks for malignancy. This is primarily due to varying rates of disease progression, as the propensity for malignancy is mostly related to degree of immunosuppression (409). Since HIV-2 progresses much more slowly than HIV-1, AIDS defining conditions often take decades to manifest even without ART. Research on HIV-1 and malignancy has been more extensive due to greater prevalence and societal impact. For example, the first case of multicentric Castelman’s disease associated with KSHV in HIV-2 was not reported until 2007, well after it was identified in HIV-1 (410).

HIV associated malignancies considered AIDS defining illnesses include Kaposi Sarcoma, select non-Hodgkin lymphomas, and cervical cancer in certain populations. AIDS defining illnesses typically occur at very low CD4 counts. HPV, which most notoriously causes cervical cancer, can be oncogenic against any immunologic backdrop, but persists longer and causes more disease in HIV infected people. There has been uncertainty about whether HIV-2 may be slightly more associated with high-grade lesions and cervical cancer than HIV-1 or whether HPV type and persistence are the determining factors (411-413). Progression of HHV-8, the etiology of Kaposi Sarcoma, may be augmented in HIV-1 compared to HIV-2 (414).EBV underlies some types of non-Hodgkin lymphoma, which has been better characterized in HIV-1 versus HIV-2 (415, 416). Nonetheless, mechanisms of oncogenesis are similar and the below discussion about HIV-associated malignancies pertains to both HIV-1 and HIV-2, except as otherwise noted. As specific malignancies associated with EBV, HHV-8 and HPV have been previously addressed, this section will focus on how HIV acts at the cellular level to potentiate oncogenicity of these and other viruses, including evidence for direct oncogenesis.

PLWH are at increased risk of developing malignancies despite effective ART, demonstrating that factors other than CD4 depletion and exhaustion of lymphopoiesis contribute. Non-AIDS defining malignancies, including hepatocellular cancer, brain cancer and squamous cell carcinomas occur at increased frequency and/or severity in PLWH compared to HIV-uninfected people (417-419). These malignancies would be expected later in the course of HIV-2 due to its slower progression. HIV facilitates development of malignancy *via* dysregulation of innate immunity, persistent immune activation, dysfunction of the inflammatory response and immunosenescence (420, 421). Causes of abnormal immune activation and inflammation are multifactorial, related to persistent antigen stimulation by viral proteins and microbial translocation, inadequate generation of memory cells, and exhaustion of effector cells (422). B and T cell defects along with aberrant monocyte and natural killer cell function perpetuate the dysfunction (423-425). Accordingly, CD4:CD8 ratio has been associated with cancer risk amongst PLWH (426).

Chronic immune activation and dysregulation, reflected by elevated inflammatory markers (427), enhance oncogenesis through several mechanisms. Since HIV replicates in activated CD4 cells, replication and spread increase. CD4 activation also upregulates CCR5 co-receptor expression, enhancing susceptibility to HIV infection (428). Impaired immune surveillance simultaneously occurs due to HIV-induced CD4 lymphopenia and ineffective CD8 responses (429, 430). CD4 lymphopenia engenders inadequate production of high-affinity antibodies, and is associated with high-risk HPV progression (431). T-cell senescence reflected by reduced T-cell polyfunctionality and T-cell receptor diversity portend poorer prognosis in certain HIV-associated malignancies (432). Ongoing inflammation is associated with HIV disease progression (433, 434) and non-AIDS comorbidities (435). These mechanisms underly the role of immunotherapy, including ART, check point inhibitors, monoclonal antibodies to specific cell types, IL-7, IL-12, and IL-15 in management of HIV-associated malignancies (436).

In addition to immunomodulatory effects, HIV proteins cause direct oncogenesis through induction of oxidative stress, integration into cancer associated and cell cycle regulatory genes, and interaction with other oncogenic viruses. The HIV proteins tat, gp120, nef, reverse transcriptase, and matrix protein p17 have been implicated in specific interactions that provoke cellular transformation, tumor propagation, and reactive oxygen species (437). For example, HCV and HIV synergistically increase reactive oxygen species in stem cells and hepatocytes. This promotes activation of kinases controlling cell growth, differentiation and apoptosis, which stimulate factors promoting inflammation and cell death, ultimately potentiating hepatocellular cancer (438). Furthermore, proviruses in CD4 cells from individuals on long-term ART can integrate into cell growth regulatory genes to influence persistence and clonal expansion of HIV-infected cells (439). A study of 534 HIV integration sites and 63 adjacent HIV env sequences from three individuals on ART for 11.3-12.7 years demonstrated identical viral sequences integrated at the same position in multiple cells consistent with infected-cell proliferation. Integrations were overrepresented in cancer-associated genes (440).

HIV may also promote oncogenesis *via* enhancement of age-related clonal hematopoiesis, which is associated with hematologic malignancies (441-443). In a prospective cohort study of 220 HIV-1 + and 226 HIV− participants ≥55 years, clonal hematopoiesis occurred in 28.2% of HIV-1+ participants and 16.8% of HIV-participants (p = 0.004); the adjusted odds ratio for occurrence of clonal hematopoiesis in HIV was 2.16 (95% CI 1.34-3.48, p = 0.002). Clonal hematopoiesis and HIV-1 infection were independently associated with inflammatory biomarkers, suggesting a selective advantage for emergence of clonal hematopoiesis in the context of chronic HIV and associated inflammation (444).

HIV not only enhances oncogenesis but may also be impacted by malignancy. Concurrent cancers can induce cytokines that activate cells (445) containing HIV proviral reservoirs. This may enhance production of replication competent virus and defective viral proteins, which stimulate the inflammatory cascade. Cancer treatments may also interact with ART, leading to reduced efficacy or impaired CD4 recovery and function (446, 447). Thus malignancy and HIV act synergistically to promote both diseases.

### Future directions

Preventing new HIV infections, identifying existing infections, effectively treating PLWH and developing strategies to mitigate the oncogenic effects of HIV will reduce the impact of HIV-associated malignancies. These approaches require augmentation of existing infrastructure in SSA and targeted public health programs, including addressing differences in HIV-1 versus HIV-2. As problem-based visits often take priority over routine health maintenance in resource limited settings, opportunities for prevention and screening of the general healthy population are limited. Point of care HIV testing and education should be offered at least once to all presenting for medical care, with regular testing for at-risk populations. Mobile screening, education of local opinion leaders and increasing awareness about prevention strategies could help reduce transmission. For PLWH, cancer screening should be standardized, and infrastructure should be developed to support treatment. National registries to ensure continuity of care should also be established, while protecting confidentiality.

Prevalence of HIV associated malignancies in SSA will reflect control of the HIV epidemic. In high-income countries, the proportion of HIV-related deaths due to cancer is increasing but has transitioned toward non-AIDS defining cancers in conjunction with improving ART coverage and HIV control at cancer diagnosis. While assessment of trends in resource limited settings is more difficult due to lack of infrastructure and data, they will likely follow the same pattern as better control of HIV is achieved and cancer screening expands (448). A 2020 review of clinical trials for treatment and prevention of HIV-associated malignancies in SSA found 11 studies specific to PLWH (449). Several trials were under the auspices of the National Cancer Institute supported AIDS Malignancy Consortium, which continues to expand efforts. AIDS Malignancy Consortium research areas for Africa include hematologic malignancies, HPV, Kaposi’s sarcoma and solid tumors (450). Expansion of such multi-national research networks will be key to addressing the situation in SSA. Future studies should fully characterize the epidemiology of HIV associated malignancies, investigate pathogenic mechanisms, and evaluate treatment, prevention, and screening strategies. Table 7 shows HIV associated malignancies, affected populations, and approaches to prevention and treatment of associated malignancies.

## Conclusions

Malignancies related to viral infection are increasing in SSA, where prevalence of viral infections is elevated compared to other regions. All six viruses classified as oncogenic (EBV, HBV, HCV, KSHV, HTLV-1 and HPV) per the International Agency for Research on Cancer impact the region. HIV-1, for which SSA has the greatest global burden, has also been linked to increasing risk of malignancy. Public health approaches to prevention of infection, such as vaccination, safer injection practices, screening of blood products, antimicrobial treatments and safer sexual practices could reduce the burden of cancer in Africa.

To combat viral associated malignancies in SSA, policy makers must prioritize public health programs that increase awareness, provide prevention resources, offer screening, and administer treatment. Cultural nuances and socioeconomic constraints must be considered during implementation of control programs. Contemporary lifestyle practices, including diet and activity patterns, will need to be addressed. Social stigma associated with malignancy will need to be overcome to facilitate discourse and encourage engagement with available resources. National level infection and cancer registries will be helpful for elucidation of local epidemiology and targeting of efforts.

We anticipate substantial increases in the burden of virally mediated cancers will occur over the next decade. It is unclear if this will be due to true increases in incidence or better detection strategies. Additional research should be conducted to characterize the dynamic epidemiology of viral oncogenesis more fully, optimize intervention strategies and accommodate behavioral-social nuances related to implementation of public health programs in SSA.

## Figures and Tables

**TABLE 1:** HBV.

Transmission	Risk Factors	Disease Course	Vaccine Target Populations	Other Control Strategies
Perinatal Parenteral Sexual	Maternal HBV infection High endemicity area Risky sexual practices Injection drug use Healthcare worker Skin piercing and tattoos	Acute infection, which may self-resolve Chronic infection, which can progress to cirrhosis or liver cancer Asymptomatic disease	Infants and children Health care workers Other high-risk groups including drug users, HIV+ individuals and those with underlying liver disease	Safe sex practices Skin piercing and tattooing safety Blood product screening Needle exchange Education of healthcare workers Treatment of HBV infected patients

**TABLE 2:** HCV.

Transmission	Risk Factors	Disease Course	Control Strategies
Perinatal Parenteral Sexual	Maternal HCV infection High endemicity area Injection drug use Risky sexual practices Healthcare worker Skin piercing and tattoos	Acute infection, which may self-resolve Chronic infection, which can progress to cirrhosis or liver cancer Asymptomatic disease	Treatment of HCV infected patients Education of healthcare workers Blood product screening Needle exchange Safe sex practices Skin piercing and tattooing safety No vaccine available

**TABLE 3:** EBV.

Transmission	Risk Factors	Example Malignancies	Control Strategies
Saliva (primary) Sex Blood Organ transplant	Geography Close contact Blood transfusion Organ transplant Immunosuppression Fomites	Burkitt’s Lymphoma Hodgkin Lymphoma Nasopharyngeal Carcinoma Post-transplant Lymphoproliferative Disorder T-cell Lymphoma B-Lymphoproliferative Disease T/NK cell Lymphoma Primary Effusion Lymphoma Gastric Carcinoma	No vaccine available No specific prevention strategies recommended No specific control strategies recommended Management depends on specific malignancy and patient factors

**TABLE 4:** HPV.

Transmission	Risk Factors	Disease Course	Vaccine Target Populations	Other Control Strategies
Sexual Skin Contact	Sex – vaginal, anal, and oral Skin contact	Asymptomatic infection Can clear spontaneously (immune response) Can progress to cervical, vulvar, vaginal, penile, anal, or oropharyngeal cancer	Males and females prior to sexual debut Given at older ages in some risk populations Recommended at ages 9-14 for females by WHO (451)	Safe sex practices Screening programs Monitoring of detected abnormalities Treatment of pre-malignant and malignant lesions

**TABLE 5:** HTLV-1 infection in humans.

Transmission	Risk Factors	Malignancies	Control Strategies
Vertical, esp. breastmilk Sex Blood Organ transplant	Geographic Breast-feeding Close contact Blood transfusion Organ transplant Intravenous drug use	Adult T-cell Leukemia/Lymphoma (ATL)	No vaccine available Screening of blood and organ donations Management of ATL depends on clinical status

**TABLE 6:** HHV-8.

Transmission	Risk Factors	Malignancies	Control Strategies
Saliva (primary) Sex Blood Organ transplant *Routes of transmission not fully understood	HHV-8 Infection Geographic Close contact Blood transfusion Organ transplant Malignancy Immunosuppression HHV-8 genotype	Kaposi Sarcoma (most common) KS-Associated Herpesvirus Inflammatory Cytokine Syndrome Multicentric Castleman’s Disease Primary Effusion Lymphoma Diffuse Large B-cell Lymphoma Germinotropic Lymphoproliferative Disorder	No vaccine available No specific prevention strategies recommended No specific control strategies recommended Management depends on specific malignancy and patient factors

**TABLE 7:** HIV-associated malignancies.

AIDS Defining Cancers	Kaposi sarcoma (HHV-8), select non-Hodgkin lymphomas (EBV), and cervical cancer (HPV)
Non-AIDS Defining Cancers	Cancers more likely to occur in HIV+ people include liver cancer, lung cancer, anal cancer, oropharyngeal cancer, and Hodgkin lymphoma. Besides HIV, HPV, HBV, and HCV increase risk.
Key Affected Populations in SSA	Females, Commercial sex workers, MSM, IDU, migrant workers, marginalized populations
Prevention Strategies	Prevention of new HIV infections, Early identification and suppressive treatment of existing HIV infections, Standardization of malignancy screening in HIV care
Treatment	Management depends on the malignancy. Cancer specific treatment programs should be accessible through HIV care providers.
